# Work Done at Fort Presidio by the Dental Corps

**Published:** 1902-10-15

**Authors:** J. S. Marshall


### WORK DONE AT FORT PRESIDIO BY THE DENTAL CORPS.

BY J. S. MARSHALL, D.D.S.

So far as the necessity is concerned of dental surgeons in the army, I think it is great. I have had occasion during the last two days to go over my records for the nine months that I have been located here at the Presidio. We have given 3,452 sittings during the nine months to the officers and entlisted men and the female nurses. We have made somewhere between 5,000 and 6,000 operations during the time.

Understand, I have with me in my office four hospital corps men. One of them is a graduate of the Toronto Dental College. The other man has had one year in college, and I learned afterwards that he had had about five years in some of these cheap John offices as an operator. I have another man who acts as clerk and keeps the records, and a fourth man who assists me individually at my chair. There are three chairs going all the time from 9 o'clock in the morning until 4 o'oclock in the afternoon, with an hour or an hour and a quarter intermission. We are doing every day just as much work as it is possible for three men to do. I had an idea that there would be a time when the work would let up but we are just as busy to-day as we were when we first started in. I could keep five men busy at the Presidio. We have something like 5,000 troops all told, that is, in camp, in quarters and in the hospital. More than that, all the outlying posts in this department come to the Presidio for their dental care. So I do not expect we shall ever find the time when we shall have a let up in the amount of work we have to do. It would probably astonish you somewhat if you should look over the records and see the character of the diseases which come to us. We see a good many diseases, I presume, that you do not see at all, some that I had never seen before—diseases which are peculiar to tropical climates. Most of these men we have are men that have come home from the Philippines, and they have inflammatory conditions of the mouth that I have never seen before, and I have had quite a large experience with hospital work, where the poor of great cities congregate for the treatment of oral conditions. I have seen nothing like it. Scores of men have come to us with a condition of the mouth like this (showing). Almost always an accompaniment of either chronic diarrhoea or dysentery. They call it in the islands, screw. It is an ulcerated condition of the gum and mucous membrane of the mouth. It commences at the festoons and then sweeps in both directions, following the gum line, and will traverse the whole mouth. The teeth become loose, but no other evidences of what we might call the symptoms of scurvy. These cases I find, in the majority of them, have a considerable amount of salivary deposit on the roots around the neck of the teeth. After that has been thoroughly removed and the gums cauterized with nitrate of silver, using a 25 percent solution, being careful of course not to allow it to spread over the mucous membrane, except where I want it. I use a little cotton and things of that kind to protect it. I find that after three or four treatments, these treatments being every day, and giving a prescription for hydrogen peroxide, using a tablespoonful to half a glass of water, and I also give a prescription for listerine to be used in the same strength, every two or three hours while they are awake, in ten days or two weeks' time I find their conditions improved, and a great many of them entirely cured.

There is another phase about that—when I first arrived at the Presidio I saw a great number of cases suffering from dysentery, diarrhoea, and found also on examination that they had this form of sore mouth. I suggested to the commanding officer of the examining hospital that as soon as these men were able to get on their feet and get up to my office he should send them and allow me to put their mouths in as good a hygienic condition as I could, to see what the result would be. The result has been very marked. In a very few weeks, three or four of these men who could not get out of the hospital for six months, some of them for eight months, with dysentery and diarrhoea, got well. I claim that the condition of the mouth, because the men could not keep their mouths and teeth clean, aggravated the dysentery or diarrhoea, and kept it up. I found if their mouths were thoroughly cleansed and put in this hygienic condition, improvement began at once.—*Pacific Gazette.*

